# Asymptomatic carriage of *Neisseria meningitidis*, *Haemophilus influenzae*, *Streptococcus pneumoniae*, Group A *Streptococcus* and *Staphylococcus aureus* among adults aged 65 years and older

**DOI:** 10.1371/journal.pone.0212052

**Published:** 2019-02-08

**Authors:** Maria Drayß, Heike Claus, Kerstin Hubert, Katrin Thiel, Anja Berger, Andreas Sing, Mark van der Linden, Ulrich Vogel, Thiên-Trí Lâm

**Affiliations:** 1 Institute for Hygiene and Microbiology, National Reference Centre for Meningococci and *Haemophilus influenzae*, University of Wuerzburg, Wuerzburg, Germany; 2 Bavarian Health and Food Safety Authority, National Consulting Laboratory for Diphtheria, Oberschleißheim, Germany; 3 Institute of Medical Microbiology, National Reference Centre for Streptococci, University Hospital (RWTH), Aachen, Germany; Ross University School of Medicine, DOMINICA

## Abstract

**Objective:**

The aim of this study was to determine the prevalence of *Neisseria meningitidis*, *Haemophilus influenzae*, *Streptococcus pneumoniae*, group A *Streptococcus* (GAS), and *Staphylococcus aureus* in asymptomatic elderly people and to unravel risk factors leading to colonization.

**Methods:**

A multi-centre cross-sectional study was conducted including 677 asymptomatic adults aged 65 years or more, living at home or in nursing homes. Study areas were Greater Aachen (North-Rhine-Westphalia) and Wuerzburg (Bavaria), both regions with medium to high population density. Nasal and oropharyngeal swabs as well as questionnaires were collected from October 2012 to May 2013. Statistical analysis included multiple logistic regression models.

**Results:**

The carriage rate was 1.9% ([95%CI: 1.0–3.3%]; 13/677) for *H*. *influenzae*, 0.3% ([95%CI: 0–1.1%]; 2/677) for *N*. *meningitidis* and 0% ([95% CI: 0–0.5%]; 0/677) for *S*. *pneumoniae* and GAS. *Staphylococcus aureus* was harboured by 28.5% of the individuals ([95% CI: 25.1–32.1%]; 193/677) and 0.7% ([95% CI: 0.2–1.7%]; 5/677) were positive for methicillin-resistant *S*. *aureus*. Among elderly community-dwellers colonization with *S*. *aureus* was significantly associated with higher educational level (adjusted OR: 1.905 [95% CI: 1.248–2.908]; *p* = 0.003). Among nursing home residents colonization was associated with being married (adjusted OR: 3.367 [1.502–7.546]; *p* = 0.003).

**Conclusion:**

The prevalence of *N*. *meningitidis*, *H*. *influenzae*, *S*. *pneumoniae* and GAS was low among older people in Germany. The *S*. *aureus* rate was expectedly high, while MRSA was found in less than 1% of the individuals.

## Introduction

Invasive infections caused by *N*. *meningitidis*, *H*. *influenzae*, *S*. *pneumoniae*, group A streptococcu*s* (GAS) and *S*. *aureus* are an important cause of morbidity and mortality in elderly people. Invasive disease due to these pathogens is more frequent at the extremes of age [1–5]. In Germany, the incidence of invasive *H*. *influenzae* disease is highest in older people (incidence 2.8/100.000 in persons aged older than 69 years) [6, 7]. The incidence of invasive meningococcal disease is low among older German adults (0.2/100.000), but the rate of septicaemia (65.1%) is highest in this age group [8]. Concerning invasive infections with MRSA, the incidence is highest in individuals aged more than 79 years (13/100.000) and 84% of the cases concern persons aged 60 years or older [7]. Moreover, elderly individuals are rather prone to have a fatal outcome, which is reflected by high case fatality rates in this age group [1, 4, 9–11]. Colonization of the skin and mucosa is considered an important source of infection, as it often precedes disease [12]. However, data on carriage rates in elderly people are scarce or lacking–notably with regard to meningococci, *H*. *influenzae* and GAS. Moreover, risk factors leading to colonization have not been well characterized. Besides vaccination against influenza, diphtheria and tetanus, the German Robert Standing Committee on Vaccination recommends immunization with the 23-valent pneumococcal polysaccharide vaccine (PPSV23) for individuals aged 60 years and more. Vaccination against *H*. *influenzae serotype b* or *N*. *meningitidis* is not part of the vaccination schedule for elderly people and is recommended only to risk groups [13]

Carriage studies on elderly people have concentrated on *S*. *aureus* in different settings such as long-term care facilities or screenings at hospital admission [14–17]. Pneumococcal carriage has been investigated in elderly individuals living at home or in care facilities in other European countries [18–20]. In Germany, however, data only existed for nursing home residents [17]. Studies on carriage of *N*. *meningitidis* have focused on children and adolescents [21] since these age groups represent a main reservoir of colonization, or on epidemiological hot spots such as the African meningitis belt [22]. Colonization with *H*. *influenzae* has also been investigated mainly in infants and children [23, 24] for the same reasons. With regard to GAS, prevalence data on asymptomatic colonization have been available for infants or children [25] and also to a smaller extend for elderly people living in long term care facilities [17, 26].

The main objective of this work was to investigate the prevalence of asymptomatic nasal or oropharyngeal carriage of *N*. *meningitidis*, *H*. *influenzae*, regardless whether typeable or not, *S*. *pneumoniae*, group A *Streptococcus* and *S*. *aureus* in persons aged 65 years and older. In addition, we aimed to identify possible risk factors leading to colonization. We therefore focused on elderly individuals living in nursing homes as well as on community-dwellers to cover all age groups and living circumstances. In this context, nasopharyngeal carriage of *Corynebacterium* sp. has also been assessed and reported previously [27].

## Materials and methods

### Study design and recruitment

The carriage study was designed and conducted by the German National Reference Centre for Meningococci and *H*. *influenzae* (NRZMHi) at the University of Wuerzburg, the German National Reference Centre for Streptococci (GNCRS) at the RTHW Aachen University, and the German National Consulting Laboratory for Diphtheria at the Bavarian Health and Food Safety Authority. Study centres were the Wuerzburg region (Bavaria) and the greater Aachen region (North Rhine Westphalia) in Germany. The German National Consulting Laboratory for Diphtheria also investigated asymptomatic carriage of Corynebacterium species, which has been published elsewhere [27]. We addressed community-dwellers attending cafés or clubs for older people as well as residents of sheltered housing and care facilities. Furthermore, a random sample of 1000 individuals stratified by age and sex, drawn from the registration office in Wuerzburg, was invited by letter. This was flanked by information campaigns via local newspapers, radio and television. An additional study group comprised geriatric inpatients of a rehabilitation hospital in Wuerzburg. Eligible participants were aged 65 years or more and not bedridden. Exclusion criteria were body temperature above 38.5°C, current infectious diseases and antibiotic therapy in the previous four weeks. To avoid seasonal bias the study period was restricted to October 2012 to May 2013. Both a nasal and an oropharyngeal swab were taken. Sampling and interviews on possible risk factors were carried out by trained staff of both reference centres. Each participant was given written information about the study. Inter-laboratory test comparisons were conducted beforehand to ensure comparability of pre-analytic and analytic processing of samples in the laboratories involved. Due to apparent prevalence differences carriage of *N*. *meningitidis* and *H*. *influenzae* was only investigated in the study area of Wuerzburg.

#### Questionnaire

The questionnaire comprised the following socio-demographic data: age, sex, marital status, educational level (high school diploma “Abitur” versus primary or secondary education) and the participant’s profession exercised the longest time before retirement. Body weight and height were recorded to calculate the body mass index (BMI). We also screened for hospitalization in the past six months, underlying medical conditions, implanted or invasive medical devices and wearing a complete or a removable partial denture (RPD). Furthermore, we asked for lifestyle habits like smoking, contact with children of preschool age, living with indoor pets, travelling abroad during the previous twelve months or presence of household members who had travelled abroad in the past twelve months. If available, the certificate of vaccination (“yellow card”) was checked for immunization against tetanus and diphtheria, *H*. *influenzae* type b (Hib), pneumococcal and meningococcal disease.

#### Ethics statement

The study protocol was approved by the ethics committee at the Medical Faculty of the University of Wuerzburg (2012-05-25; reg.-no. 150/11). Each participant or legal representative gave informed written consent.

### Laboratory procedures

One single nasal swab was taken from both anterior nares and an oropharyngeal swab was taken from the posterior pharyngeal wall. Swabs were inserted into Amies transport medium (MASTASWAB, MAST Diagnostica GmbH, Reinfeld, Germany), stored at room temperature and processed within 4 hours [28]. Nasal samples were plated on Columbia agar with 5% sheep blood (bioMérieux Deutschland GmbH, Nürtingen, Germany), while oropharyngeal swabs were plated on a meningococcal selective agar first (modified Martin-Lewis Agar, BD, Heidelberg, Germany), then on blood agar and a selective agar for *Haemophilus* species (BD Chocolate Agar with IsoVitaleX and Bacitracin, BD). Agar plates were incubated at 35 ± 2°C in 5% CO_2_ for 48 hours and screened for suspect colonies after 24 hours and 48 hours. Colonies were subcultured on blood agar or–in case of presumed *Haemophilus* species–on chocolate agar (GC II Agar with IsoVitaleX, BD). Preliminary identification was based on characteristic growth on selective media and gram stain. At least five different morphologies of colonies growing on chocolate agar with bacitracin were analysed by Matrix Assisted Laser Desorption/Ionization Time of Flight Mass Spectrometry (MALDI-TOF MS; VITEK-MS, bioMérieux Deutschland GmbH) to exclude *H*. *parainfluenzae*. Concerning suspected pneumococci an optochin test was done on at least five different alpha haemolytic colonies from each blood agar plate analyzed. For staphylococcal identification, catalase and latex agglutination test (Pastorex Staph Plus, Bio-Rad Laboratories GmbH, Munich, Germany) was carried out. All suspected isolates were stored at—80°C for final species confirmation and further analysis. This was performed on *N*. *meningitidis* as described by Claus et al. [29], and on *S*. *pneumoniae* and GAS as reported by Imöhl et al. [30, 31]. Species confirmation of all non-*Haemophilus parainfluenzae* isolates included sequencing of the ompP6 gene [32] and multilocus sequence typing (MLST) [33]. Sequence types were assigned using the PubMLST-database (https://pubmlst.org/hinfluenzae/). Antibiotic susceptibility patterns of *S*. *aureus* strains were determined using VITEK 2 automated systems (bioMérieux).

### Statistical analysis

Proportions were calculated for categorical data. Median, interquartile range (IQR) and total range were determined for the continuous variable “age”, but this variable was additionally transformed into a categorical variable by clustering in three age groups (65–74, 75–84 and 85 years and older). BMI was classified as “normal weight or underweight” for values less than 25 and “overweight or obesity” for values greater than or equal 25. Differences between categorical data were compared using two-sided χ^2^ or Fisher’s exact test as appropriate [34] and exact instead of asymptotic *p*-values. Multi-variable logistic regression analysis was applied as backward regression with a cut-off *p*-value < 0.05 for entry into the model. Variables with *p*-values < 0.25 [35] were selected for analysis as well as the variables study region and sex. *P*-values < 0.05 were considered significant. Odds ratios and prevalence data were calculated with 95% confidence intervals (CI). For prevalence data with low values the Clopper and Pearson exact method was used. IBM SPSS statistics version 23 was used for all calculations.

## Results

In total, 785 volunteers participated, 57 individuals had to be excluded mainly due to prior antibiotic intake. The remaining 728 participants comprised 531 (72.9%) community-dwellers, 146 (20.1%) nursing home residents, and 51 (7.0%) inpatients of a geriatric rehabilitation hospital in Wuerzburg. The latter group was assessed separately. Among the 677 community-dwellers and nursing home residents, 203 (30.0%) individuals were living in Greater Aachen (North-Rhine-Westphalia), and 474 (70.0%) participants in the Wuerzburg region (Bavaria).

The median age of all participants was 78 years (IQR: 11 years, total range: 65–106 years), 30.6% (207/677) were males and 69.4% (470/677) were females. Concerning the education level 22.9% of the individuals had a high school diploma (155/677), of whom only two participants had been occupied as a worker. A subset of 213 (31.5%) participants provided their international certificate of vaccination. Most of those individuals (88.7%; 189/213) were vaccinated against diphtheria and tetanus (Td). Only 42.3% (n = 90) were vaccinated against pneumococcal disease. With one exception, all participants had received the 23-valent pneumococcal polysaccharide vaccine. Two individuals were (0.9%) vaccinated against meningococcal disease and Hib respectively. With regard to *N*. *meningitidis*, only the tetravalent conjugate vaccine against the serogroups A, C, W-135, Y was used in the study period. Concerning the date of the last Td vaccination, we observed a time period of one year or less in 14.8% (27/182) and a time period of more than 10 years in 12.1% (22/182) of the participants with fully completed certificate of vaccination. The last pneumococcal vaccination was administered within the last year in 12.0% (10/84) of the individuals and more than 6 years ago in 30.8% (26/84). Dates of vaccination against meningococci and Hib were not available.

The median age of the 51 geriatric inpatients was 83 years (IQR: 14 years, range: 65–98 years), 25.5% were males (n = 13). As mentioned above, these participants were assessed separately (prevalence data and risk factors) since they differed from the general population in terms of underlying conditions.

*H*. *influenzae* was found in 1.9% ([95%CI: 1.0–3.3%]; 13/677) of the study participants. The median age was 73 years (IQR: 10 years; total range: 65–89 years). The prevalence was 2.4% (n = 5) among males and 1.7% (n = 8) among females (*p* = 0.550). Carriage rates in community-dwellers (2.1%; n = 11) and residents of nursing homes (1.4%; n = 2) did not differ significantly (*p* = 0.745). One strain was serotype e, the remaining 12 strains were nontypeable *H*. *influenzae* (NTHi). All isolates were β-lactamase negative and susceptible to ampicillin. Among three carriers with certificate of vaccination, none reported immunization against Hib. We did not observe a higher prevalence among individuals with contact to children of preschool age (2.3% or 4/175; *p* = 0.751).

The prevalence of *N*. *meningitidis* was 0.3% ([95%CI: 0–1.1%]; 2/677). Both of the 76 and 73 year-old carriers were female community-dwellers and had no contact with children of preschool age. One of them was co-colonized with NTHi. GAS and *S*. *pneumoniae* were detected in none of the participants ([95% CI: 0–0.5%]; 0/677). An overview on prevalence data is given in the supplemental material **S1 Table**.

### *S*. *aureus*

*S*. *aureus* was found in 28.5% of all participants ([95% CI: 25.1–32.1%]; 193/677) with a slightly higher colonization rate of 31.5% in residents of nursing homes ([24.1–39.7%]; 46/146) compared to 27.7% ([23.9–31.7%]; 147/531) among community-dwellers (*p* = 0.408; χ^2^ test, not significant). Detailed information about prevalence data of *S*. *aureus* and MRSA is given in **Table 1**.

**Table 1:** Prevalence of *S*. *aureus* and MRSA.

	*S*. *aureus*	MRSA
	n (% [95%CI])	n (% [95%CI])
Total participants* n = 677	193 (28.5 [25.1–32.1])	5 (0.7 [0.02–1.7])
Community-dwellers* n = 531	147 (27.7 [23.9–31.7])	2 (0.4 [0–1.4])
Nursing home residents* n = 146	46 (31.5 [24.1–39.7])	3 (2.1 [0.4–5.9])
Geriatric inpatients n = 51	16 (31.4 [19.1–45.9])	1 (2.0 [0–10.4])

MRSA, methicillin-resistant *S*. *aureus*; CI, confidence interval.

*Geriatric inpatients excluded.

Five of 677 individuals (0.7% [95% CI: 0.02–1.7%]) harboured methicillin-resistant *S*. *aureus* (MRSA; 2.60% of all *S*. *aureus* isolates; age range: 73–88 years). In contrast to community-dwellers (0.4% [95% CI: 0–1.4%]; 2/531) MRSA was found more frequently in nursing homes (2.1% [95% CI: 0.4–5.9%]; 3/146; *p* = 0.07; Fisher’s exact test). The carriage rate was also elevated in elderly people with diabetes mellitus (2.50% or n = 3; *p* = 0.04; Fisher’s exact test) and in participants with a prosthetic joint (2.70% or n = 3; *p* = 0.033; Fisher’s exact test). Only one carrier reported inpatient treatment.

Among the 51 geriatric inpatients the prevalence of *S*.*aureus* was 31.4% ([95% CI: 19.1–45.9%]; n = 16). MRSA was detected in one inpatient only.

#### Risk factors for colonization with *S*. *aureus*

Risk factors for colonization were analysed separately for elderly community-dwellers and for residents of nursing homes, as both subgroups differed in terms of living circumstances and recruitment strategy (information via local media and random sampling versus visiting selected care facilities). Males were colonized more frequently both in total (32.9%; n = 68; *p* = 0.116; χ^2^ test) and in all subgroups, though not significantly (Tables 2 and 3). Regarding age, hospitalization, BMI, comorbidities and lifestyle habits differences in carriage rates were small or did not reach significance (data reported in Tables 4 and 5). The prevalence of *S*. *aureus* was significantly higher in community-dwelling subjects with a high school diploma (38.0% or 49/129; *p* = 0.003; Table 2). Participants living in nursing homes were colonized more frequently if they were married (*S*. *aureus* prevalence: 40.5%; *p* = 0.007; Table 3) and if they had a gastrostomy / PEG-tube (80.0% or 4/5; *p* = 0.034). Geriatric inpatients with joint prostheses showed a significantly higher carriage rate (48.10% or 13/27; *p* = 0.008, χ^2^ test) compared to patients with other conditions.

**Table 2:** Prevalence of *S*. *aureus* in elderly community-dwellers by socio-demographic factors.

Variable		Total participants^a^	Presence of *S*. *aureus*	*p*-value^b^
		n (%)	n (%)	
Total		531 (100)	147 (27.7)	
**Socio-demographic data**				
Sex	Male	168 (31.6)	53 (31.5)	0.211
	Female	363 (68.4)	94 (25.9)	
				
Age [years]	65–74	219 (41.2)	60 (27.4)	0.991
	75–84	229 (43.1)	64 (27.9)	
	≥85	83 (15.6)	23 (27.7)	
Marital status	Married	189 (35.6)	43 (22.8)	0.068
Educational level	High school diploma	129 (24.3)	49 (38.0)	**0.003**
Profession	University graduate	18 (3.4)	7 (38.9)	0.289
	Self-employed	25 (4.7)	9 (36.0)	0.362
	Ordinary or medium level civil servant	24 (4.5)	5 (20.8)	0.496
	Senior or higher civil servant	60 (11.3)	19 (31.7)	0.540
	Employee–non-executive	191 (36.0)	44 (23.0)	0.086
	Executive employee	78 (14.7)	25 (32.1)	0.411
	Un- or semi-skilled worker	29 (5.5)	10 (34.5)	0.523
	Skilled worker or master craftsman	42 (7.9)	13 (31.0)	0.719
	Housewife	58 (10.9)	13 (22.4)	0.358
	Others / not applicable	6 (1.1)	2 (33.3)	0.671

^a^Elderly individuals living at home or in sheltered accommodation.

^b^Comparison by carriage using χ^2^ or Fisher's exact test and *p* < 0.05 as the cut-off for significance; significant results in bold.

**Table 3:** Prevalence of *S*. *aureus* in nursing home residents by socio-demographic factors.

Variable		Total participants^a^	Presence of *S*. *aureus*	*p*-value^b^
		n (%)	n (%)	
Total		146 (100)	46 (31.5)	
**Socio-demographic data**				
Sex	Male	39 (26.7)	15 (38.5)	0.316
	Female	107 (73.3)	31 (29.0)	
				
Age [years]	65–74	10 (6.8)	3 (30.0)	0.959
	75–84	55 (37.7)	18 (32.7)	
	≥85	81 (55.5)	25 (30.9)	
Marital status	Married	84 (57.5)	34 (40.5)	**0.007**
Educational level*	High school diploma	26 (18.1)	9 (34.6)	0.642
Profession	University graduate	3 (2.1)	0 (0)	0.552
	Self-employed	3 (2.1)	1 (33.3)	1.0
	Ordinary or medium level civil servant	6 (4.1)	1 (16.7)	0.665
	Senior or higher civil servant	7 (4.8)	4 (57.1)	0.207
	Employee–non-executive	41 (28.1)	9 (22.0)	0.165
	Executive employee	15 (10.3)	4 (26.7)	0.776
	Un- or semi-skilled worker	25 (17.1)	11 (44.0)	0.159
	Skilled worker or master craftsman	8 (5.5)	2 (25.0)	1.0
	Housewife	36 (24.7)	12 (33.3)	0.837
	Others / not applicable	2 (1.4)	2 (100)	0.098

^a^Nursing home residents.

^b^Comparison by carriage using χ^2^ or Fisher's exact test and *p* < 0.05 as the cut-off for significance; significant results in bold.

*Data not available for 1.4% of the study population.

**Table 4:** Prevalence of *S*. *aureus* in elderly community-dwellers and health and lifestyle factors.

Variable		Total participants^a^	Presence of *S*. *aureus*	*p-*value^b^
		n (%)	n (%)	
Total		531 (100)	147 (27.7)	
Study region	AAC	152 (28.6)	37 (24.3)	0.286
	WUE	379 (71.4)	110 (29.0)	
**Health status**				
BMI	<25	220 (41.4)	69 (31.4)	0.116
	≥25	311 (58.6)	78 (25.1)	
Inpatient treatment in past 6 months*		77 (14.6)	19 (24.7)	0.583
Diabetes mellitus		79 (14.9)	26 (32.9)	0.277
Heart failure		107 (20.2)	34 (31.8)	0.333
Asthma		29 (5.5)	6 (20.7)	0.409
Cancer		40 (7.5)	9 (22.5)	0.472
Hypertension		253 (47.6)	71 (28.1)	0.923
Atopic dermatitis		14 (2.6)	5 (35.7)	0.547
Implanted or invasive device^c^		108 (20.3)	27 (25.0)	0.548
Gastrostomy		0 (0)	NA	NE
Pacemaker		13 (2.4)	5 (38.5)	0.362
Joint prosthesis		83 (15.6)	21 (25.3)	0.689
Denture		274 (51.6)	80 (29.2)	0.439
Complete denture		127 (23.9)	37 (29.1)	0.733
Removable partial denture		172 (32.4)	49 (28.5)	0.836
**Lifestyle**				
Smoking*		21 (4.0)	8 (38.1)	0.318
Contact with children of preschool age*		148 (28.0)	35 (23.6)	0.196
Living with indoor pets*		64 (12.1)	19 (29.7)	0.766
Travel abroad in past 12 months*		158 (29.8)	47 (29.7)	0.525
International travel of household members in past 12 months		88 (16.6)	29 (33.0)	0.241

AAC, Aachen; WUE, Wuerzburg; BMI, body mass index; NA, not applicable; NE, non executable.

^a^Elderly individuals living at home or in sheltered accommodation.

^b^Comparison by carriage using χ^2^ or Fisher's exact test and *p* < 0.05 as the cut-off for significance; significant results in bold.

^c^Including gastrostomy, tracheostomy, ventilation, central venous catheter, urinary catheter, dialysis, pacemaker, joint prosthesis.

*Missing data (<1% of the study population).

**Table 5:** Prevalence of *S*. *aureus* in nursing home residents and health and lifestyle factors.

Variable		Total participants^a^	Presence of *S*. *aureus*	*p*-value^b^
		n (%)	n (%)	
Total		146 (100)	46 (31.5)	
Study region	AAC	51 (34.9)	13 (25.5)	0.269
	WUE	95 (65.1)	33 (34.7)	
**Health status**				
BMI	<25	80 (57.1)	24 (30.0)	0.715
	≥25	60 (42.9)	20 (33.3)	
Inpatient treatment in past 6 months*		35 (24.3)	13 (37.1)	0.533
Diabetes mellitus		40 (27.4)	12 (30.0)	0.845
Heart failure		50 (34.2)	15 (30.0)	0.852
Asthma		7 (4.8)	2 (28.6)	1.0
Cancer		16 (11.0)	5 (31.3)	1.0
Hypertension		50 (34.2)	15 (30.0)	0.852
Atopic dermatitis		2 (1.4)	1 (50.0)	0.532
Implanted or invasive device^c^		44 (30.1)	19 (43.2)	0.054
Gastrostomy		5 (3.4)	4 (80.0)	**0.034**
Pacemaker		5 (3.4)	2 (40.0)	0.650
Joint prosthesis		28 (19.2)	11 (39.3)	0.368
Denture		113 (77.4)	37 (32.7)	0.672
Complete denture		65 (44.5)	19 (29.2)	0.720
Removable partial denture		50 (34.2)	17 (34.0)	0.708
**Lifestyle**				
Smoking		8 (5.5)	2 (25.0)	1.0
Contact with children of preschool age*		27 (18.8)	10 (37.0)	0.495
Living with indoor pets		7 (4.8)	2 (28.6)	1.0
Travel abroad in past 12 months*		2 (1.4)	1 (50.0)	0.535
International travel of household members in past 12 months*		4 (2.8)	2 (50.0)	0.589

AAC, Aachen; WUE, Wuerzburg; BMI, body mass index.

^a^Residents of retirement and nursing homes.

^b^Comparison by carriage using χ^2^ or Fisher's exact test and *p* < 0.05 as the cut-off for significance; significant results in bold.

^c^Including gastrostomy, tracheostomy, ventilation, central venous catheter, urinary catheter, dialysis, pacemaker, joint prosthesis.

*Up to 1.4% of data not available.

In the multivariable logistic regression model analyzing elderly community-dwellers the variable “High school diploma” was significantly associated with carriage (OR: 1.911 [95% CI: 1.252–2.918]; *p* = 0.003), (**Table 6**). Residents of nursing homes were at risk if they were married (OR: 3.367 [95% CI: 1.502–7.546]; *p* = 0.003), were former semi- or unskilled workers (OR: 2.701 [95% CI: 1.028–7.096]; *p* = 0.044) or had an implanted or invasive device (OR: 2.591 [95% CI: 1.169–5.744]; *p* = 0.019). Interestingly, participants providing their yellow card exhibited a significantly lower colonization rate (21.6% or 46 of 213; *p* = 0.008) with similar trends in all subgroups. However, this finding was not included in our regression models, since it was not considered a risk factor but rather a marker for other behavioural traits.

**Table 6:** Multivariable logistic regression models: Risk factors associated with carriage of *S*. *aureus*.

Subgroup	Variable	Odds Ratio [95% CI]	*p*-value
**Community-dwellers**^**a**^	High school diploma	1.911 [1.252–2.918]	**0.003**
**Nursing home residents**^**b**^	Being married	3.367 [1.502–7.546]	**0.003**
	Implanted or invasive device^c^	2.591 [1.169–5.744]	**0.019**
	Un- or semi-skilled worker	2.701 [1.028–7.096]	**0.044**

CI, confidence interval

^a^Model adjusted for the variables study area, sex, being married, high school diploma, non-executive employee, BMI ≥ 25, contact with children of preschool age, international travel of household members in past 12 months.

^b^Model adjusted for the variables study area, sex, being married, un- or semi-skilled worker, implanted or invasive device.

^c^Including gastrostomy, tracheostomy, ventilation, central venous catheter, urinary catheter, dialysis, pacemaker, joint prosthesis.

## Discussion

The main finding of our study was a remarkably low prevalence of the four bacteria investigated. Only *S*. *aureus* was detected in higher rates, whereas colonization with MRSA was rare. To our knowledge, this is the first study to investigate carriage rates of *N*. *meningitidis* and *H*. *influenzae* in elderly individuals. Besides residents of nursing homes our study also included elderly community-dwellers, as prevalence data on colonization with GAS are lacking in this group. In Germany, the colonization rate of *S*. *pneumoniae* was also unknown for elderly individuals living at home.

The overall prevalence of *S*. *aureus* (28.5%) is in line with the results of other studies [17, 36, 37] according to which the German population shows higher colonization rates compared to other European countries [38]. MRSA was rare among community-dwellers (0.4%), similar to investigations in other parts of Germany [37]. The prevalence was also rather low among nursing home residents compared with other surveys, where carriage rates ranged from 3.7% and 5.5% in Brazilian and German long-term care facilities to 15.7% in Spanish and 17.2% in Italian nursing homes [39–43]. The assessment of risk factors for colonization had been a further objective of this study. In terms of health-associated factors, our results indicate that older people with invasive or implanted devices, such as a PEG tube, a urinary catheter but also a joint prosthesis, are at risk. These individuals often lack mobility and may need a higher amount of support and care. More frequent health care contacts consequently, in combination with the fact that a device is implanted, which might serve as a matrix to which bacteria adhere, might explain higher carriage rates [14, 17, 44]. The MRSA prevalence was higher among residents of nursing homes and significantly higher among elderly individuals with a prosthetic joint or diabetes. This is in line with other findings and highlights the importance of effective hygiene measures especially in long-term care facilities [45, 46].

Concerning socio-demographic characteristics we did not ask participants for known risk factors such as working in the health care sector or occupational contact to livestock [37, 47], but aimed to gain information about their socio-economic status. We observed an association between colonization with *S*. *aureus* and variables that suggest the presence of close household contacts. Married residents of nursing homes had a higher risk for carriage, which has also been found in other studies [48]. Being married of course does not necessarily mean cohabiting or sharing a room in a care facility. Nevertheless, this at least implicates having cohabited during a certain period of life. Former occupation as a semi- or un-skilled worker, another risk factor in our study, can be regarded as a marker for lower income. This often includes crowded dwelling conditions, i.e. higher numbers of persons per room or lack of bedrooms, and thus seems to be plausible, too [37]. In contrast to a previous study [45] elderly individuals living at home–a study group with younger age cohorts (see also tables)—were more likely to be colonized with *S*. *aureus* if they had obtained a high school diploma. Further research is necessary to identify reasons for this observation. However, our results confirm the role of socio-economic risk factors for *S*. *aureus* carriage, which should be taken into account e.g. in screening programs.

*S*. *pneumoniae* was detected in none of the participants. In fact, colonization rates strongly depend on sampling site, preanalytic conditions, processing methods (cultural or molecular detection by PCR) and age. Hence, colonization rates in elderly individuals can range from 0% [49] to 22% [19]. There is no clear evidence from the data available which sampling site yields optimal detection rates in adults. If swabbing only one site instead of sampling both nasopharynx and oropharynx as recommended by the WHO, a missing rate of up to 45% has to be accepted [50]. Nevertheless, we decided for pragmatic reasons not to collect nasopharyngeal samples, as we expected a better compliance to sampling notably among older and more anxious participants. Another limitation could be the lack of using a pneumococcal selective agar, which makes it more difficult to identify suspect colonies among other alpha haemolytic species. We restricted the plating of one oropharyngeal swab on few different agar plates to avoid losing potential isolates. In light of the above, it is unclear whether the pneumococcal carriage rate was underestimated in this study.

Our study presents the first meningococcal carriage rates in healthy individuals aged 65 years and older. Colonization with *N*. *meningitidis* turned out to be very rare (0.3%) in this age group. This is in good agreement with other findings. An Australian study recently reported a colonization rate of 0.32% among a smaller population of geriatric inpatients [51]. Our group conducted a carriage study of *N*. *meningitidis* among adolescents and adults using direct plating, which avoids a loss of bacteria by inserting swabs into a transport medium [52]. A recent review suggests that swabs should be taken from the posterior pharyngeal wall, which was followed in this study [28]. Furthermore, the authors suggest that placing swabs into transport medium is equivalent to direct plating, if the swabs are plated out within 5 h, which again was the practice in our study. Of course, a single swab in its nature is limited in sensitivity [53]. We nevertheless assume that carriage rates are very low among elderly people >65 years, which adds to the scarce information on this subject available from the literature. None of the carriers had reported contact to children. However, we did not systematically search for contact to adolescents or younger adults, a risk group showing the highest carriage rates [21], which is a limitation of this study.

The carriage rate of *H*. *influenzae* was also low (1.9%) among elderly individuals. Almost all isolates were true NTHi, apart from one Hi-e strain, as shown by molecular methods. Thus, our results correlate well with present trends showing that NTHi has become the most important *H*. *influenzae* type in invasive disease [54]. Since the agar plate selective for *haemophilus* species was the third plating, one cannot exclude, whether this could have reduced the detection rate. However, since we observed extremely low carriage rates of *N*. *meningitidis* (first plating) while the carriage rate was higher for *H*. *influenzae*, we presume that the order of plating probably did not have a strong impact on the detection rate of *H*. *influenzae*. Future research should also include longitudinal studies to better understand transmission dynamics of NTHi in the elderly. Furthermore, the sensitivity of single swabs needs to be addressed carefully, e.g. by application of PCR technology.

None of our participants harboured GAS. A German study on nursing home residents previously reported a carriage rate of 3.2% for beta haemolytic streptococci [17]. According to a meta-analysis on outbreak reports of invasive GAS disease in care facilities [26] carriage rates can vary between 0% and 16.4%. However, investigating a care environment related to invasive cases is a particular setting, which makes it difficult to compare with our study population, notably with elderly community-dwellers. To our knowledge, reliable data on GAS carriage in elderly people living at home are lacking. We nevertheless presume that colonization with GAS is very rare in this group.

Our prevalence data on *S*. *pneumoniae*, *N*. *meningitidis*, *H*. *influenzae* and GAS provide insight into carriage rates of potentially pathogenic commensals in the elderly population. Both study areas, situated in the west and the south of Germany, represent geographical regions with medium population density surrounded by countryside areas, which is quite characteristic for Germany [55, 56]. However, prevalence data might differ from large cities like Berlin or Hamburg with different social stratification, which should be considered for future studies. Limitations to the study are the lack of longitudinal data and potentially low sensitivity as in the case of *S*. *pneumoniae*. The study elucidates risk factors for *S*. *aureus* carriage, which emphasize the role of social activity and medical conditions. Although we were able to recruit a large cohort of individuals, the results are limited by the heterogeneity of the cohort comprising community-dwelling individuals as well as persons living in nursing homes. This heterogeneity was inherent to the fact that recruitment of volunteers in this age group is a challenge. Future studies should apply longitudinal study designs and in addition use PCR technology for detection.

## Supporting information

S1 Table Prevalence of *H*. *influenzae* and *N*. *meningitidis*. (DOCX)

S2 Table Questionnaire–original questions. (DOCX)
