# Correction: Asymptomatic carriage of *Neisseria meningitidis*, *Haemophilus influenzae*, *Streptococcus pneumoniae*, Group A *Streptococcus* and *Staphylococcus aureus* among adults aged 65 years and older

**DOI:** 10.1371/journal.pone.0305830

**Published:** 2024-06-14

**Authors:** The PLOS ONE Staff

The seventh author’s initials appear incorrectly in the citation of PubMed. The correct citation is: Drayß M, Claus H, Hubert K, Thiel K, Berger A, Sing A, van der Linden M, Vogel U, Lâm TT. Asymptomatic carriage of Neisseria meningitidis, Haemophilus influenzae, Streptococcus pneumoniae, Group A Streptococcus and Staphylococcus aureus among adults aged 65 years and older. PLoS One. 2019 Feb 8;14(2):e0212052. doi: 10.1371/journal.pone.0212052. PMID: 30735539; PMCID: PMC6368330.

The publisher apologizes for the error.

## References

[1] Drayß M, Claus H, Hubert K, Thiel K, Berger A, Sing A, et al. (2019) Asymptomatic carriage of *Neisseria meningitidis*, *Haemophilus influenzae*, *Streptococcus pneumoniae*, Group A *Streptococcus* and *Staphylococcus aureus* among adults aged 65 years and older. PLOS ONE 14(2): e0212052. 10.1371/journal.pone.0212052 30735539 PMC6368330

