# Supplementary material for: Asymptomatic carriage of Neisseria meningitidis, Haemophilus influenzae, Streptococcus pneumoniae, Group A Streptococcus and Staphylococcus aureus among adults aged 65 years and older
Source: PLoS One. 2019 Feb 8;14(2):e0212052. doi: 10.1371/journal.pone.0212052 (PMC6368330; doi:10.1371/journal.pone.0212052)
Supplement: S1 Table — (DOCX) [file pone.0212052.s001.docx]

**S 1. Prevalence of *H. influenzae* and *N. meningitidis.***

|  | ***H. influenzae*** | ***N. meningitidis*** |
| --- | --- | --- |
|  | n (% [95%CI]) | n (% [95%CI]) |
| Total participants* n=677 | 13 (1.9 [1.0-3.3]) | 2 (0.3 [0-1.1]) |
| Community-dwellers* n=531 | 11 (2.1 [1.0-3.7]) | 2 (0.4 [0-1.4]) |
| Nursing home residents* n=146 | 2 (1.4 [0.2-4.9]) | 0 (0 [0-2.5]) |
| Geriatric inpatients n=51 | 0 (0 [0-7.0]) | 1 (2.0 [0-10.4]) |

CI, confidence interval.

*Geriatric inpatients excluded.
