# Supplementary material for: Asymptomatic carriage of Neisseria meningitidis, Haemophilus influenzae, Streptococcus pneumoniae, Group A Streptococcus and Staphylococcus aureus among adults aged 65 years and older
Source: PLoS One. 2019 Feb 8;14(2):e0212052. doi: 10.1371/journal.pone.0212052 (PMC6368330; doi:10.1371/journal.pone.0212052)
Supplement: S2 Table — (DOCX) [file pone.0212052.s002.docx]

**S2. Questionnaire – original questions**

| Deutsch | | English | |
| --- | --- | --- | --- |
| Probandenfragebogen | | **Questionnaire for participants** | |
|  |  |  |  |
| Alter [Jahre]: | | **Age [years]:** |  |
| Körpergröße [cm]: | | **Body height [cm]:** | |
| Körpergewicht [kg]: | | **Body weight [kg]:** | |
|  |  |  |  |
| Geschlecht: | Männlich | **Sex:** | Male |
|  | Weiblich |  | Female |
|  |  |  |  |
| Familienstand: | Ledig | **Marital status:** | Single |
|  | Verheiratet |  | Married |
|  | Verwitwet |  | Widowed |
|  | Getrennt lebend / geschieden | | Living separately / divorced |
|  |  |  |  |
| 1. Wurden Sie innerhalb der letzten 6 Monate im Krankenhaus behandelt? | | **1. Have you been treated in hospital within the last 6 months?** | |
|  | Nein |  | No |
|  | Innerhalb der letzten 4 Wochen | | Within the last 4 weeks |
|  | Innerhalb der letzten 3 Monate | | Within the last 3 months |
|  | Innerhalb der letzten 6 Monate | | Within the last 6 months |
|  |  |  |  |
| 2. Liegen bei Ihnen eine der folgenden Erkrankungen vor? | | **2. Do you have one of the following medical conditions?** | |
|  | Diabetes mellitus | | Diabetes mellitus |
|  | Herzinsuffizienz | | Heart failure |
|  | Asthma |  | Asthma |
|  | Krebs/Tumorerkrankung | | Cancer |
|  | Bluthochdruck |  | High blood pressure |
|  | Atopische Dermatitis | | Atopic dermatitis |
|  | Keine der genannten Erkrankungen | | None of the medical conditions above |
|  |  |  |  |
| 3. Liegen bei Ihnen eine der folgenden medizinischen Maßnahmen vor? | | **3. Do you have one of the following medical measures** | |
|  | Gastrostoma / PEG | | Gastrostomy / PEG |
|  | Tracheostoma |  | Tracheostomy |
|  | Blasenkatheter |  | Urinary catheter |
|  | Beatmung |  | Ventilation |
|  | Zentralvenöser Katheter / Port | | Central venous catheter / port |
|  | Dialyse |  | Dialysis |
|  | Herzschrittmacher | | Pacemaker |
|  | Gelenkprothese | | Joint prosthesis |
|  | Keine der genannten Maßnahmen | | None of the medical measures above |
|  |  |  |  |
| 4. Fehlt bei Ihnen die Milz (angeboren, nach chirurgischer Entfernung oder Unfall) oder funktioniert Ihre Milz nicht (funktionelle Asplenie)? | | **4. Is your spleen missing (congenital, after surgical removal or an accident) or is your spleen not working (functional asplenia)?** | |
|  | Ja |  | Yes |
|  | Nein |  | No |
|  |  |  |  |
| 5. Tragen Sie eine Zahnprothese? | | **5. Do you wear a removable denture?** | |
|  | Nein |  | No |
|  | Ja, eine Zahnvollprothese (ohne Halt an verbleibenden Zähnen) | | Yes, a complete denture (denture not fixed on teeth) |
|  | Ja, eine Zahnteilprothese (mit Halt an verbleibenden Zähnen) | | Yes, a removable partial denture (denture held in place by remaining teeth) |
|  |  |  |  |
| 6. Welche Aussage trifft auf Ihre Rauchgewohnheiten zu? | | **6. Which statement applies to your smoking habits?** | |
|  | Ich bin Nicht-Raucher | | I am a non-smoker |
|  | Ich bin Raucher |  | I am a smoker |
|  | Ich bin ehemaliger Raucher und habe seit mehr als 6 Monate mit dem Rauchen aufgehört | | I am a former smoker and have stopped smoking for more than 6 months |
|  | Ich bin ehemaliger Raucher und habe seit weniger als 6 Monate mit dem Rauchen aufgehört | | I am a former smoker and have stopped smoking for less than 6 months |
|  |  |  |  |
| 7. Falls Sie Raucher sind, wie viele Zigaretten rauchen sie pro Tag? | | **7. If you are a smoker, how many cigarettes do you smoke per day?** | |
|  | Weniger als eine Zigarette pro Tag | | Less than one cigarette per day |
|  | 1 bis 5 Zigaretten pro Tag | | 1 to 5 per day |
|  | 6 bis 10 Zigaretten pro Tag | | 6 to 10 per day |
|  | 11 bis 20 Zigaretten pro Tag | | 11 to 20 per day |
|  | Mehr als 20 Zigaretten pro Tag | | More than 20 per day |
|  |  |  |  |
| 8. Haben Sie Kontakt mit Kindern im Vorschulalter? | | **8. Do you have contact with children of preschool age?** | |
|  | Ja |  | Yes |
|  | Nein |  | No |
| Falls Ja: Wie oft haben Sie Kontakt mit Kindern im Vorschulalter? | | **If yes: how frequent do you have contact with children of preschool age?** | |
|  | Mehr als zweimal pro Woche | | More than twice a week |
|  | Etwa ein- bis zweimal pro Woche | | Around once or twice a week |
|  | Etwa 2-3 Mal pro Monat | | Around 2 to 3 times a month |
|  | Etwa 4-12 Mal pro Jahr | | Around 4 to 12 times a year |
|  | Etwa 2-3 Mal pro Jahr | | Around 2 or 3 times a year |
|  | Nicht mehr als einen Tag im Jahr | | Not more than one day a year |
|  |  |  |  |
| 9. Haben Sie Haustiere? | | **9. Do you keep indoor pets?** | |
|  | Ja |  | Yes |
|  | Nein |  | No |
| Falls Ja: Welche der folgenden Haustiere haben Sie? | | **If yes: which of the following pets do you have?** | |
|  | Hund |  | Dog |
|  | Katze |  | Cat |
|  | Vogel |  | Pet bird |
|  | Meerschwein |  | Guinea pig |
|  | Hamster |  | Hamster |
|  | Hase / Kaninchen | | Rabbit |
|  | Zierfische |  | Ornamental fish |
|  |  |  |  |
| 10. Sind Sie in den letzten 12 Monaten ins Ausland gereist? | | **10. Have you travelled abroad during the last 12 months?** | |
|  | Ja |  | Yes |
|  | Nein |  | No |
| Falls ja, waren Sie in einem der folgenden Länder? | | **If yes, have you been in one of the following countries?** | |
|  | Russland |  | Russia |
|  | Weißrussland |  | Belarus |
|  | Moldawien |  | Moldova |
|  | Ukraine |  | Ukraine |
|  | Armenien |  | Armenia |
|  | Aserbaidschan |  | Azerbaijan |
|  | Georgien |  | Republic of Georgia |
|  | Kasachstan |  | Kazakhstan |
|  | Kirgistan |  | Kyrgyzstan |
|  | Tadschikistan |  | Tajikistan |
|  | Turkmenistan |  | Turkmenistan |
|  | Usbekistan |  | Uzbekistan |
|  | Estland |  | Estonia |
|  | Lettland |  | Latvia |
|  | Littauen |  | Lithuania |
|  | Indien |  | India |
|  | Pakistan |  | Pakistan |
|  | Nepal |  | Nepal |
|  | Indonesien |  | Indonesia |
|  | Philippinen |  | Philippines |
|  | Brasilien |  | Brazil |
|  | Haiti |  | Haiti |
|  | Dominikanische Republik | | Dominican Republic |
|  |  |  |  |
| 11. Leben bei Ihnen Personen im Haushalt, welche in den letzten 12 Monaten ins Ausland gereist sind? | | **11. Are there household members, who have travelled abroad during the last 12 months?** | |
|  | Ja |  | Yes |
|  | Nein |  | No |
| Falls ja, waren diese in einem der folgenden Länder? | | **If yes, have they been in one of the following countries?** | |
|  | Russland |  | Russia |
|  | Weißrussland |  | Belarus |
|  | Moldawien |  | Moldova |
|  | Ukraine |  | Ukraine |
|  | Armenien |  | Armenia |
|  | Aserbaidschan |  | Azerbaijan |
|  | Georgien |  | Republic of Georgia |
|  | Kasachstan |  | Kazakhstan |
|  | Kirgistan |  | Kyrgyzstan |
|  | Tadschikistan |  | Tajikistan |
|  | Turkmenistan |  | Turkmenistan |
|  | Usbekistan |  | Uzbekistan |
|  | Estland |  | Estonia |
|  | Lettland |  | Latvia |
|  | Littauen |  | Lithuania |
|  | Indien |  | India |
|  | Pakistan |  | Pakistan |
|  | Nepal |  | Nepal |
|  | Indonesien |  | Indonesia |
|  | Philippinen |  | Philippines |
|  | Brasilien |  | Brazil |
|  | Haiti |  | Haiti |
|  | Dominikanische Republik | | Dominican Republic |
|  |  |  |  |
| 12. Was ist Ihr höchster allgemeinbildender Schulabschluss? | | **12. What is your highest degree or level of school you have completed?** | |
|  | Ich bin von der Schule gegangen ohne Abschluss | | I left school without any degree. |
|  | Ich habe den Hauptschulabschluss (Volkschulabschluss) | | I finished elementary school |
|  | Ich habe den Realschulabschluss (Mittlere Reife) | | I finished secondary school |
|  | Ich habe den Abschluss der Polytechnischen Oberschule | | I finished polytechnic secondary school |
|  | Ich habe die Fachhochschulreife | | I attained "Fachhochschulreife" (=high school exam with restriction to certain colleges) |
|  | Ich habe die allgemeine oder fachgebundene Hochschulreife / Abitur | | I attained "Abitur" (German equivalent to high school diploma) |
|  |  |  |  |
| 13. Welchen Beruf haben Sie am längsten ausgeübt? | | **13. Which profession did you exercise the longest time before retirement?** | |
|  | Ich war Akademiker in freiem Beruf (Arzt, Rechtsanwalt, Steuerberater o.ä.) | I was a university graduate, self-employed (physician, lawyer, tax accountant, or similar) and had | |
|  | Keine weiteren Mitarbeiter | | No staff |
|  | 1 bis 4 Mitarbeiter | | 1-4 staff members |
|  | 5 und mehr Mitarbeiter | | 5 or more staff members |
| Ich war selbständig im Handel, Gewerbe, Handwerk, Industrie, Dienstleistung, bzw. PGH-Mitglied | | I was self-employed, engaged in trade, craft, industry, service, or worked as PGH-member (PGH= cooperative of craft, a manufacturing association in the former German Democratic Republic) | |
|  | Keine weiteren Mitarbeiter | | No staff |
|  | 1 bis 4 Mitarbeiter | | 1-4 staff members |
|  | 5 und mehr Mitarbeiter | | 5 or more staff members |
|  | PGH-Mitglied |  | PGH-member |
| Ich war Beamter, Richter, Berufssoldat | | I was a civil servant, judge, regular soldier, … | |
|  | Einfacher Dienst | | Ordinary civil service |
|  | Mittlerer Dienst | | Medium-level civil service |
|  | Gehobener Dienst | | Senior civil service |
|  | Höherer Dienst |  | Higher civil service |
| Ich war Angestellter | | I worked as an employee | |
|  | Mit ausführender Tätigkeit nach Anweisung (z.B. Verkäufer) |  | With subordinate task according to instructions (e.g. shop assistant) |
|  | Mit Tätigkeit nach Anweisung (z.B. Krankenschwester) |  | With task according to instructions (e.g. Nurse) |
|  | Mit selbständiger Leistung in verantwortlicher Tätigkeit (z.B. Prokurist) |  | With limited executive function (e.g. authorized officer) |
|  | Mit umfassenden Führungsaufgaben (z.B. Geschäftsführer) |  | With executive function (e.g. CEO) |
| Ich war Arbeiter | | I was a worker |  |
|  | Ungelernt |  | Unskilled |
|  | Angelernt |  | Semi-skilled |
|  | Facharbeiter |  | Skilled |
|  | Vorarbeiter |  | Foreman |
|  | Meister |  | Master craftsman |
| Keine feste Tätigkeit | | No special business | |
|  | Hausfrau |  | I was a housewife |
|  | Freiberuflicher Künstler / Musiker | | Freelancing artist / musician |
|  |  | Not applicable |  |
|  |  |  |  |
|  |  |  |  |
| Mitarbeiterfragebogen | | **Staff questionnaire** | |
|  |  |  |  |
| Wohnsituation des Probanden: | | **Participant's type of dwelling:** | |
|  | Zu Hause lebend | | Community-dwelling |
|  | Betreutes Wohnen | | Sheltered housing |
|  | Pflegeheim |  | Care home |
|  |  |  |  |
| Ort: |  | **Place:** |  |
|  |  |  |  |
| 1. Hat der Teilnehmer eine der folgenden Erkrankungen oder Therapien? | | **1. Does the participant have one of the following conditions or therapies?** | |
|  | Fieber (Körpertemperatur >= 38°C), | | Fever (body temperature equal or above 38°C), |
|  | Laufende antibiotische Therapie | | current antibiotic therapy |
|  | Ja |  | Yes |
|  | Nein |  | No |
|  |  |  |  |
| 2. Nimmt der Proband an einer weiteren wissenschaftlichen Studie teil? | | **2. Is the participant involved in further scientific studies?** | |
|  | (Falls ja, Ausschlusskriterium!) | | (If yes, exclusion criterium!) |
|  | Ja |  | Yes |
|  | Nein |  | No |
|  |  |  |  |
| 3. Hatte der Teilnehmer eine antibiotische Therapie während der letzten 4 Wochen? | | **3. Has the participant been under antibiotic therapy for the last 4 weeks?** | |
|  | Ja |  | Yes |
|  | Nein |  | No |
|  | Weiß nicht |  | Do not know |
|  |  |  |  |
| 4. Liegt der Impfausweis des Teilnehmers vor? | | **4. Did the participant show his certificate of vaccination?** | |
|  | Ja |  | Yes |
|  | Nein |  | No |
|  |  |  |  |
| 4_1 | **Impfung gegen Diphtherie und Tetanus (Td)?** | **4_1** | **Vaccination against diphtheria and tetanus (Td)?** |
|  | Ja |  | Yes |
|  | Nein |  | No |
| Datum: |  | Date: |  |
| Impfstoff: |  | Vaccine: |  |
|  |  |  |  |
| 4_2 | **Impfung gegen Pneumokokken?** | **4_2** | **Vaccination against pneumococci?** |
|  | Ja |  | Yes |
|  | Nein |  | No |
| Datum: |  | Date: |  |
| Impfstoff: |  | Vaccine: |  |
|  |  |  |  |
| 4_3 | **Impfung gegen Hib?** | **4_3** | **Vaccination against Hib?** |
|  | Ja |  | Yes |
|  | Nein |  | No |
| Datum: |  | Date: |  |
| Impfstoff: |  | Vaccine: |  |
|  |  |  |  |
| 4_4 | **Impfung gegen Menigokokkenmeningitis?** | **4_4** | **Vaccination against meningococci?** |
|  | Ja |  | Yes |
|  | Nein |  | No |
| Datum: |  | Date: |  |
| Impfstoff: |  | Vaccine: |  |
